# Multifunctional biocatalysis: An unusual imine reductase

**DOI:** 10.1016/j.engmic.2022.100023

**Published:** 2022-05-13

**Authors:** Feifei Chen, Jianhe Xu, Gaowei Zheng

**Affiliations:** State Key Laboratory of Bioreactor Engineering, Shanghai Collaborative Innovation Center for Biomanufacturing, East China University of Science and Technology,Shanghai 200237, China

**Keywords:** Imine reductase, Biocatalysis, Chiral amine, Conjugate reduction, Reductive amination

## Abstract

A multifunctional biocatalyst EneIRED capable of catalyzing amine-activated conjugate alkene reduction and subsequent reductive amination was discovered. The enzyme realized the coupling of α, β-unsaturated carbonyls with amines to efficiently synthesize a broad set of chiral amine diastereomers based on its unusual active site structure and catalytic mechanism.

## Graphical abstract

**Figure:**
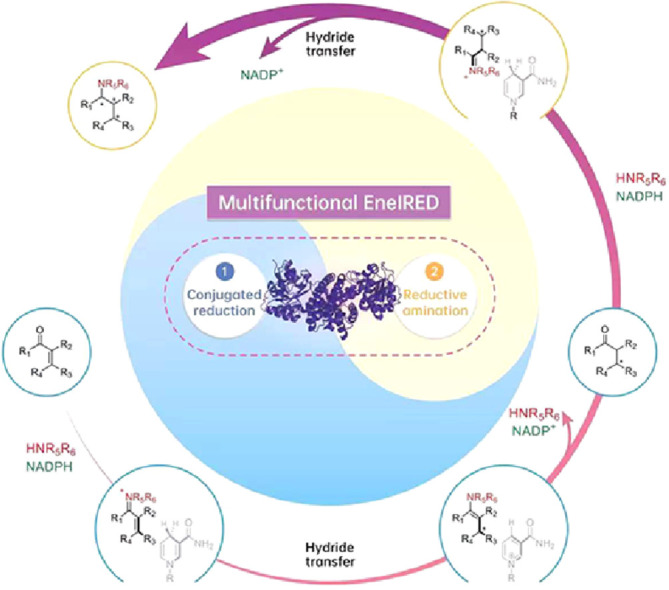

Structurally diverse chiral amines are ubiquitous structural motifs in biologically active compounds such as natural products and drugs [1,2]. The efficient synthesis of chiral amines has received significant interest, as demonstrated by the development of various synthetic routes involving diastereoisomeric crystallization, asymmetric reductive amination of prochiral ketones, reduction of imines or enimines, and C-H amination [3]. Amino compounds typically contain more than one stereogenic center, and their asymmetric synthesis with adequate (dia)stereoselectivity is challenging and usually requires tedious multistep syntheses or intricated cascade catalysis systems [4,5]. Multi-enzymatic cascades provide new opportunities for preparing amino compounds with multiple stereogenic centers, but their application is hampered by issues such as incompatibility in the reaction system, generation of byproducts, and complicated work-up [6,7]. Now, Turner and collaborators have discovered a multifunctional biocatalyst EneIRED from a panel of metagenomic imine reductases (IREDs), which can catalyze both amine-activated conjugate reduction (CR) of alkenes and following reductive amination (RA), thus achieving the coupling of α,β-unsaturated carbonyls with amines for the efficient synthesis of diverse chiral amine diastereomers (Fig. 1) [8].

**Fig. 1:**
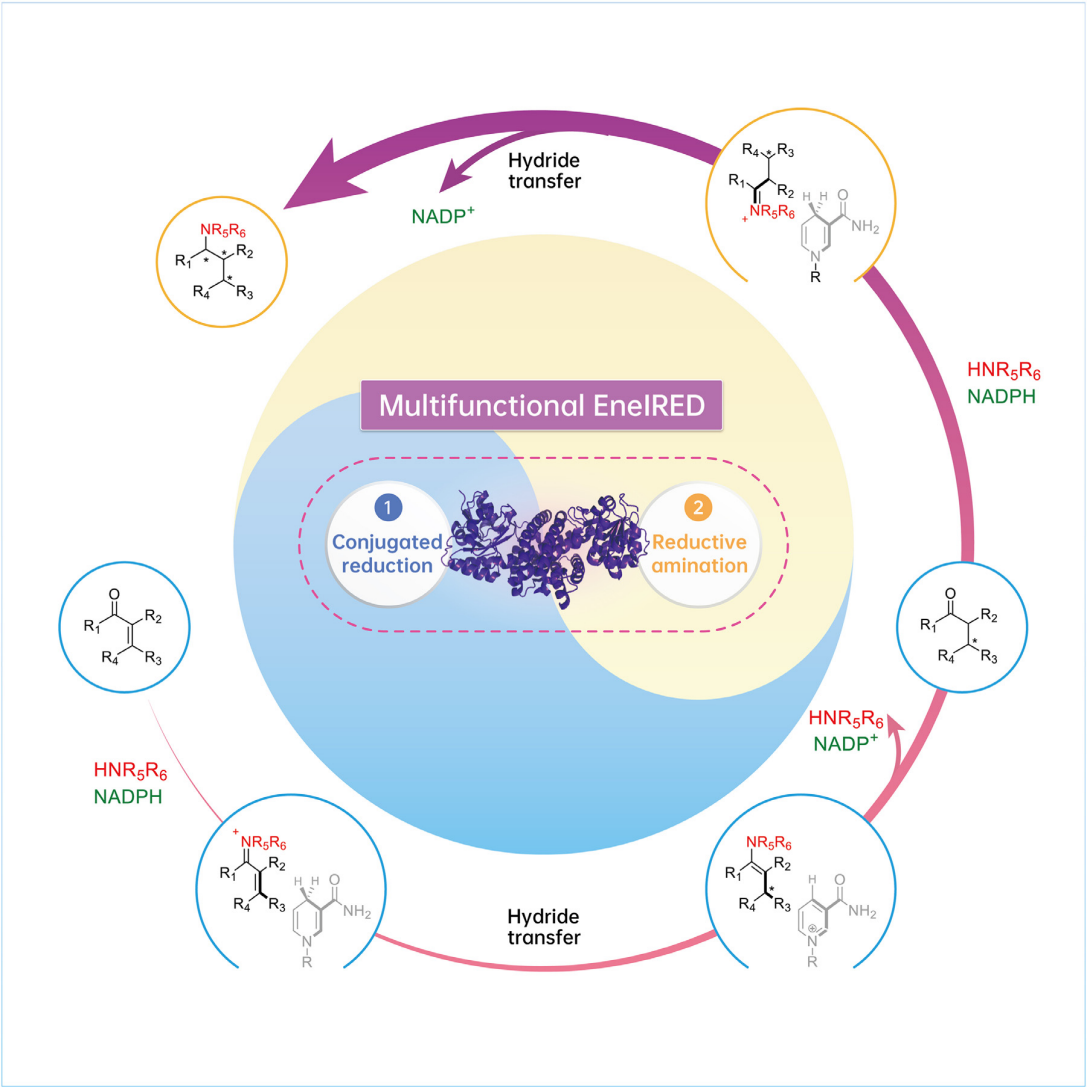
Proposed catalytic cycle of EneIRED-catalyzed CR–RA. α,β-Unsaturated carbonyls with amine partners were transformed into the corresponding stereoenriched saturated amines through EneIRED-catalyzed CR and RA. Adapted from reference [8].

IREDs are a family of NADPH-dependent enzymes and are involved in the reduction of C=N bonds and reductive amination of C=O bonds [9]. Furthermore, in a few cases, IREDs have been shown to catalyze the direct reduction of C=O bonds to form corresponding alcohol products [10]. However, the biocatalytic reduction of C=C bonds was still an unexplored area for IREDs, whereas the reaction has typically been performed with ene-reductases (EREDs) [11]. After screening a total of 389 IREDs, including both reported and recently developed (meta)genomic IREDs [12] with a cyclic ene-imine as the model substrate, 44 enzymes were found that could catalyze the asymmetric reduction of both the C=C and the C=N bonds of the model substrate. Among them, an IRED, presumably from *Pseudomonas* species (EneIRED), displayed excellent reactivity for the double reduction CR-RA.

Under optimized conditions, EneIRED exhibited a broad substrate profile, enabling CR-RA, mono-CR, or mono-RA of diverse α,β-unsaturated carbonyls with ammonia or varied amines. Less hindered enals and enones as well as amine nucleophiles tend to be converted with good chemoselectivity to the corresponding saturated amine products. For example, coupling of (*E*)-2-methyl-2-butenal with cyclopropylamine afforded >99% conversion to the saturated amine. Furthermore, unsubstituted and substituted cycloalkyl-2-enones with different ring sizes were well tolerated by the enzyme, affording good conversion and chemo- and stereoselectivity for many of the corresponding CR–RA products. In particular, C_3_-substitutioned cyclohex-2-enone substrates were efficiently transformed to CR–RA products even when coupled with different amine partners (*e.g.,* NH_3_, methylamine, allylamine, propargylamine, *n*-butanamine, pyrrolidine, and (*R*)- or (*S*)-3-fluoropyrrolidine), yielding high conversion and chemo-, enantio- and diastereoselectivity in many cases. The enzymatic CR-RA also demonstrated great synthetic applicability, with 60–81% isolated yield for the preparative synthesis of the saturated amine products. In one case, 64% isolated yield was attained at a scale of 1.0 mmol.

The unusual function of EneIRED inspired the researchers to conduct mechanistic investigations into the enzymatic catalysis. Isotopic labelling experiments using regenerated deuterated nicotinamide cofactor to transform 3-methyl-2-cyclohexenone with cyclopropylamine as an amine partner showed that hydride transfer occurs at the C-1 and C-3 positions of the carbonyl substrate. A reaction time-course study and several control experiments suggested that the enone substrate first undergoes CR to the corresponding ketone before transformation into the saturated amine via RA, and an ene-imine-NAD(P)H-biocatalyst complex could be essential for EneIRED-catalyzed CR. The researchers further resolved the structure of EneIRED to obtain structural insights into the catalytic mechanism. Consequently, structural features were observed in the active site compared to the other IREDs with released structures in the database, and residues Y177 and Y181 proved to play significant roles in the CR-RA. Molecular docking of the enzyme with the resulting ene-imine intermediate suggests that the prochiral carbon atom of the C=C bond is positioned at a suitable distance to transfer the hydride from the pyridinium ring of the cofactor. Based on mechanistic and structural studies, an EneIRED catalytic cycle of CR-RA was delineated by the researchers (Fig. 1). First, the nicotinamide cofactor NAD(P)H and the formed ene-imine intermediate from α,β-unsaturated carbonyl and amine partner are accepted in the active site of EneIRED, generating an ene-imine-NAD(P)H-enzyme complex. The hydride is then transferred from nicotinamide cofactor to the C-3 of the ene-imine due to its beneficial orientation, whereby CR occurs and generates the stereoenriched 1-enamine-NAD(P)^+^-EneIRED complex. The prochiral 1-enamine and NAD(P)^+^ are then released from the active site, and the former undergoes hydrolysis to the stereoenriched carbonyl. Subsequently, the intermediate imine is formed from the stereoenriched carbonyl and the amine partner and complexed with the enzyme and nicotinamide cofactor NAD(P)H, which is transformed into the final stereoenriched amine product via IRED-catalyzed RA.

The identification of new enzymes and new activities of previously discovered enzymes plays a significant role in extending the synthetic scope of biocatalysis and pushing forward its application in the manufacture of high-value chiral compounds [13,14]. Although the catalysis of IREDs, especially in the synthesis of chiral amines, has been widely investigated in the last decade [9,[15], [16], [17]], the identification of the multifunctional biocatalyst EneIRED provides new perspectives in the biocatalytic area due to this newly disclosed function for C=C reduction [8]. The CR-RA catalysis enabled by EneIRED in a one-pot, one-catalyst fashion provides a concise, green, and efficient route to stereoenriched amine products with multiple stereogenic centers. This is a significant discovery that improves upon previously developed methods, considering the existing issues associated with cost, operational complexity, environmental impact, and product quality [4], [5], [6], [7].

The catalytic mechanism and chemoselectivity to imine reduction, reductive amination, and ketoreduction have been widely studied for IREDs with structural insights [15,17,18]. Nevertheless, the identification of the new CR-RA function of IREDs with structural investigations sheds new light on understanding of the structure-activity relationships of the family of IREDs, which provides guidance for the laboratory evolution of the enzymes to enable the synthesis of structurally different chiral amines of interest. The study will likely inspire further exploration or creation of new enzyme activities for other NAD(P)H-dependent enzyme families (*e.g.,* amine dehydrogenases [19,20]) based on structural study and by virtue of advances in protein engineering [21].

## Declaration of Competing Interest

The authors declare that they have no known competing financial interests or personal relationships that could have appeared to influence the work reported in this paper.
